# Responding to experienced and anticipated discrimination (READ): anti -stigma training for medical students towards patients with mental illness – study protocol for an international multisite non-randomised controlled study

**DOI:** 10.1186/s12909-019-1472-7

**Published:** 2019-01-31

**Authors:** Tanya Deb, Heidi Lempp, Ioannis Bakolis, Tushar Vince, William Waugh, Claire Henderson, the INDIGO READ study group, Graham Thornicroft, Shuntaro Ando, Sosei Yamaguchi, Asami Matsunaga, Shinsuke Kondo, Kayo Ichihashi, Yasutaka Ojio, Makoto Ogawa, Chiyo Fujii, Kiyoto Kasai, Andrea Candelas, Laura Martín, Andrea Jiménez, Cristina Castañeda, Cecilia Hernández, Jesús de la Higuera, José Eduardo Muñoz-Negro, Mercedes Sola, Rocío García, José Miguel Gota, Juan Francisco Mula, Ana López, Amadeo Oria, Jorge A. Cervilla, Aguila Bono, Dolores Franco, Jaime Gómez, Carmen Jiménez, Remedios Dorado, Evelio Ingunza, Irene Márquez, Diego de la Vega, Pablo Gª-Cubillana, Uta Ouali, Lamia Jouini, Yosra Zgueb, Rabaa Jomli, Fethi Nacef, Megan Campbell, Dan Stein, Judit Harangozo, Tunde Masseyferguson Ojo, A. Ogunwale, A. O. Sowunmi, S. S. Awhangansi, Deji Ogundapo, O. T. Sodiya, Babatunde Fadipe, Andrew T. Olagunju, Adebayo R. Erinfolami, Peter O. Ogunnubi, Catarina Cardoso Tomás, Dzmitry Krupchanka, Marco Pascucci, Simon Vasseur Bacle, Antoine Colliez, Deborah Sebbane, Amaury Mengin, Pierre Vidailhet, Cyril Cazals, Alp Ucok, Andrea Fiorillo, Gaia Sampogna, Micaela Savorani, Valeria Del Vecchio, Mario Luciano, Giuseppina Borriello, Benedetta Pocai, Prince Nwaubani, Yvonne James, Andrea Tocca, Ranjan Pattnaik, Shanthi Chilasagaram, Zhang Wufang

**Affiliations:** Health Service and Population Research Department, David Goldberg Centre, King’s College London, Institute of Psychiatry, Psychology and Neuroscience, De Crespigny Park, London, SE5 8AF UK

**Keywords:** Stigma, Discrimination, Medical education

## Abstract

**Background:**

Stigma and discrimination are a significant public health concern and cause great distress to people with mental illness. Healthcare professionals have been identified as one source of this discrimination. In this article we describe the protocol of an international, multisite controlled study, evaluating the effectiveness of READ, an anti-stigma training for medical students towards patients with mental illness. READ aims to improve students’ ability to minimise perceived discriminatory behaviours and increase opportunities for patients, therefore developing the ability of future doctors to address and challenge mental illness related discrimination. READ includes components that medical education research has shown to be effective at improving attitudes, beliefs and understanding.

**Methods/design:**

READ training was developed using evidence based components associated with changes in stigma related outcomes. The study will take place in multiple international medical schools across high, middle and low income countries forming part of the INDIGO group network, with 25 sites in total. Students will be invited to participate via email from the lead researcher at each site during their psychiatry placement, and will be allocated to an intervention or a control arm according to their local teaching group at each site. READ training will be delivered solely to the intervention arm. Standardised measures will be used to assess students’ knowledge, attitudes and skills regarding discrimination in both the intervention and control groups, at baseline and at follow up immediately after the intervention. Statistical analyses of individual-level data will be conducted using random effects models accounting for clustering within sites to investigate changes in mean or percentages of each outcome, at baseline and immediately after the intervention.

**Discussion:**

This is the first international study across high, middle and low income countries, which will evaluate the effectiveness of training for medical students to respond effectively to patients’ experiences and anticipation of discrimination. The results will promote implementation of manualised training that will help future doctors to reduce the impact of mental illness related discrimination on their patients. Limitations of the study are also discussed.

## Background

Mental health related stigma and discrimination constitute a significant public health concern, leading to reduced help seeking and access to healthcare [1, 2], fewer opportunities for education and work [3, 4], and increased co-morbidity [5] and mortality [6, 7] for people with mental illness. There is a need for interventions to address this discrimination as acknowledged by international mental health policies [8, 9]. For instance in the UK mental health policy for 2011–2015 [8], one of the six key objectives of the Government’s mental health strategy specifies the need to ensure fewer people experience stigma and discrimination due to their mental illness. Internationally, the World Health Organisations’ Mental Health Action Plan 2013–2020 [9] stipulates that people affected by mental illness should be able to participate fully in society and at work, free from stigmatisation and discrimination.

Several professional groups have been identified as significant sources of stigma and discrimination. One such group is healthcare staff, including doctors and medical students [10]. As a result, health professionals have been identified as a target group by two national anti-stigma campaigns: Opening Minds in Canada [11] and One of Us in Denmark (http://www.en-af-os.dk/da/Om%20kampagnen/Fokusomraader.aspx). In addition the Andalusian Strategy Against Stigma campaign in Spain developed an action plan with several interventions for health professionals, including training courses and a video documentary (http://www.1decada4.es/course/view.php?id=12#profesionales). However, the potential for doctors to show leadership in reducing the impact of discrimination has not yet been thoroughly examined [12]. Any training on mental health related stigma and discrimination should therefore acknowledge doctors’ roles as both sources of discrimination and as potential anti-stigma change agents. To date, medical student education has done only the former [13, 14], with previous training showing short term changes in attitudes but with no focus on future anti-stigma agency in either content or assessment. Further, these projects have not made use of research in medical education for students, which has highlighted the effectiveness of critical reflection and self reflection as methods to improve attitudes, beliefs, understanding of a subject and satisfaction in learning. A number of reflective techniques used in healthcare education have been associated with these improvements, particularly critical reflection [15]. Critical reflection is defined as the emotional and intellectual activity through which people critically assess content, and process information to interpret and give meaning to an experience [16]. Critical reflection is part of a model of learning called reflective practice, which has been frequently used in healthcare education over the last decade [15, 17]. In addition reflection is shown to be positively associated with attitudes, beliefs and understanding [15]. Therefore reflection is an important component to include in any educational training for medical students.

This project implements training for medical students entitled ‘Responding to Experienced and Anticipated Discrimination’ (READ), and aims to evaluate its effectiveness by measuring changes in students’ knowledge, attitudes and skills in responding to mental illness related discrimination.

There are elements from both medical education and anti-stigma interventions to create an intervention tailored to this group, and focussed on behaviour in addition to attitudes and knowledge.

### Development of READ

The aim of READ training is to develop the role of future doctors to address and challenge mental illness related discrimination, by improving medical students’ ability to:

- increase opportunities for patients e.g. for access to health services or employment (due to patients’ anticipation of discrimination),
- respond to discrimination and apply current evidence for effective anti-stigma interventions
- minimise behaviours that may be experienced by patients as discriminatory.

The content, delivery and evaluation of READ was informed by: (i) studies of patients’ experiences of discrimination;(ii) studies of stigma and discrimination among healthcare professionals; (iii) research on contact-based education to reduce stigma in health care professionals; and (iv) the broader field of study on intergroup contact as a means to reduce prejudice. The decision to use contact based education was based on several reviews of interventions to reduce stigma and prejudice [18–21] including among young people [22] and health professionals [10], supplemented by more recent papers on the evaluation of interventions with health professionals delivered as part of Canada’s anti-stigma programme [23, 24]. The literature on patients’ experiences from which content was derived was based on a systematised search for studies on mental health related discrimination.

Patients’ experiences have been studied using both quantitative and qualitative methods. Surveys using the Discrimination and Stigma Scale (DISC) include examples of experiences provided by [25–27]. Qualitative work on subjective experiences of stigma in patients, relatives and mental health professionals provides richer data on the range of experiences patients find discriminatory [28, 29]. These examples are included in the READ presentation.

Research in both Emergency Department and general hospital settings has identified factors contributing to perceived discrimination and diagnostic overshadowing (when a physical illness is incorrectly attributed to the patient’s diagnosis of mental illness) [30, 31]. These factors and observations of discriminatory practice, witnessed by healthcare staff, are discussed in the training.

Evaluation of contact-based education delivered through the Canadian anti-stigma programme Opening Minds [11] identified the key components associated with positive stigma related outcomes. This study evaluated anti-stigma training programmes for healthcare professionals across Canada, and used qualitative and quantitative analysis to determine the key elements present in the most effective programmes. The results showed that (i) including multiple forms of contact with people with lived experience of mental illness (i.e. live and filmed), and (ii) personal accounts with a focus on recovery, were significantly associated with better outcomes on mental illness related knowledge and attitudes. READ has therefore incorporated both these key components: to include a person with lived experience of mental illness (expert by experience as educator) to co-deliver the training with a psychiatrist, and short films of testimonies by experts by experience, to ensure the training comprises multiple forms of contact. In addition the expert by experience will provide a personal account of her/his illness and recovery process, therefore ensuring the “focus on recovery” component is addressed. Finally, a previous study of anti- stigma training for medical students [14] suggested that a repeat or “booster” session might facilitate sustained improvements in mental illness related knowledge and attitudes. READ includes a first session followed by a second “booster” session, which may consequently contribute to longer term improvement in these outcomes.

READ developed using previous research on intergroup contact (as in this case healthcare professionals are one group and patients with mental illness are another group). A study in the UK [32] assessed the application of intergroup contact theory to mental health related stigma. This work examined the influence of different types of imagined contact with people with schizophrenia, and concluded that imagined contact might increase intergroup anxiety (and therefore desire for social distance) unless it was purposefully structured to reflect a positive imagined contact experience. A meta-analysis of over 500 studies [33] found that reduced anxiety and increased empathy towards the other group are key mediators of the effect of intergroup contact on prejudice. We have taken these mediators into account in both the development and evaluation of READ. It is important to note that this design generally treats the two groups as if they are dichotomous, in this case that the psychiatry trainer and medical students do not have lived experience of mental illness. However, in the second READ session the psychiatrist trainer discusses stigma in relation to the self (internalised stigma) and in relation to colleagues, including as it relates to professionalism and regulatory requirements for doctors to prevent their illness from affecting patient care.

READ delivery began in 2015 in the UK to medical students at two medical schools. These have acted as pilot teaching sites for the training components. The content and structure of the training has been revised for the proposed study, using feedback from focus groups with the participating medical students in the pilot teaching sites. Past and current experts by experience include NHS Trust employees who have accessed mental health services, and peer support workers with a diagnosis of mental illness.

## Methods/design

This is a multisite, non-randomised controlled study. The sites are 25 medical schools in 15 countries, including low, middle and high income countries, as outlined in Table 1 below. Sites were recruited via previous contact within the INDIGO Network (a collaboration of researchers in different countries co-ordinated by the Centre for Global Mental Health, King’s College London) or personal invitation to researchers interested in challenging stigma in healthcare professionals.

**Table 1:** Distribution of medical schools

	Continent	Country	Number of sites
	Europe	UK	3
		Czech Republic	1
		France	2
		Spain	3
		Italy	2
		Portugal	3
		Hungary	1
		Turkey	1
	Asia	India	2
		Japan	1
		Taiwan	1
		China	1
	Africa	South Africa	1
		Nigeria	2
		Tunisia	1
Summary	3	15	25

### Intervention

The READ intervention has been manualised to provide guidance on implementation for the sites, which includes suggestions on how to adapt the training to make the content suitable for each site’s resources and culture. The manual also includes a section on preparation and practical considerations to aid with implementing the study. It attempts to address the reduced resources in some of the sites by suggesting that sites in the same country share resources where possible, and if necessary. The sites also have the option to correspond with each other to share strategies to facilitate implementation.

READ training will be provided to students in small groups according to local teaching arrangements. Students will be able to feed back after the role play and to ask questions of the trainers.

The first session is delivered over 1.5 h near the start of the psychiatry rotation in each medical school; the second session, lasting 1 h, takes place later before the end of the rotation. The length of the psychiatry rotation varies in each medical school, however we advised at least 1 week elapses between the two sessions. This will allow time for students to identify examples of discrimination to discuss in the second session. The training is designed to also help the students interact more effectively with patients they meet during the psychiatry rotation. Therefore it is intended to enhance their overall learning and provide a useful experience rather than add unnecessarily to workload.

The first session includes:

- a personal account by an expert by experience of their illness and recovery;
- a structured presentation, co-delivered by the psychiatrist and expert by experience, on stigma and its impact on people with mental health problems;
- one or two video clips of a service user testimony. Some examples of video clips with different styles have been sent to participating sites, so that sites can then find and use appropriate clips, allowing for differences in culture and language;
- Two role plays by the students, one of each of experienced and anticipated discrimination;
- At the end of the first session students are provided with an assignment: to describe specific situations they encounter during their psychiatric rotations of anticipated or experienced discrimination reported by service users. The descriptions will then be discussed and reflected upon during the second session.

The second session consists of:

- Discussion facilitated by both trainers of any experienced or anticipated discrimination students have observed during their time meeting with patients in clinical settings;
- discussion of how to support healthcare professional colleagues with mental health problems, and possible disclosure of mental illness;
- how to respond to one’s own mental health problems

### The expert by experience as educator

Where possible service users working as educators or in peer support roles will act as the expert by experience to co-deliver the training. The presentation by the expert by experience will include a description of the onset of illness; current treatment and self-management of illness; examples of stigma and discrimination; and how the illness informs aspects of the person’s life, for example their choice of study or work. The expert by experience can also contribute their views based on the slide content during both sessions, subject to the time available. The service user will be asked to refrain from generalised criticism of health professionals or services as medical students are not responsible for these shortcomings. In addition to decreased anxiety as a result of positive contact, empathy is an important mechanism for stigma reduction [33], and if students are made to feel defensive it will be more difficult for them to empathise with the service user. The psychiatrist and expert by experience will debrief together after each session to discuss what went well and any concerns.

### Control arm

A control arm is important for two reasons. First, to control for any effect of psychiatry training that occurs between the first and second sessions; second, to control for the possible effect of the baseline measures (see below). The control group will therefore be administered all the measures at the same times as the intervention group, i.e. at two sessions during which the intervention group is receiving the training. Alternatively, two separate, consecutive psychiatry rotations may act as the intervention and control groups, if this is feasible. Students will be allocated by the trainer to either the control or the intervention group according to their usual local teaching group allocation at each site.

### Measures

Mental health-related knowledge will be measured by the Mental Health Knowledge Schedule [MAKS] [34]. The MAKS comprises six items that cover stigma-related mental health knowledge areas: help seeking, recognition, support, employment, treatment, and recovery, and six items regarding classification of various conditions as mental illnesses. The scale has been tested and found to have good psychometric properties [34]. The total score is calculated so that higher MAKS scores indicate greater mental health knowledge.

Attitudes to mental illness and psychiatry will be measured with the Mental Illness Clinicians’ Attitudes (MICA2) scale [35]. This 16-item scale was developed with input from mental health service users, carers, medical students and trainee psychiatrists, and tested with medical students. The scale assesses stigmatising attitudes to both people with mental illness and to the medical speciality of psychiatry. Psychometric testing of the scale showed good internal consistency and test-retest reliability [35].

Skills will be assessed via an observed structured clinical examination (OSCE) where a student interacts with a simulated patient for 7 minutes in the presence of an examiner [36]. Participating simulated patients will be given a briefing sheet prior to the OSCE to standardise their role and responses to students. The OSCE was developed to assess behaviour and communication skills in consultation with a clinical-medical education expert and lead in Clinical Assessments at King’s College London (TV). It consists of a clinical scenario in which each medical student will discuss a formal referral to a local mental health team for treatment and support of the service user’s psychosis. The simulated patient will report experienced and anticipated discrimination. The student is expected to verbally acknowledge the encountered and reported situation by the service user, demonstrate an empathic attitude (verbally expressed/body language), and explore with the service user her/his concerns. The student will be assessed on (i) their response to the reports of anticipated and experienced discrimination; (ii) the extent to which they acknowledge the stereotypes of people with psychosis and distinguish these from the actual diagnosis and proposed treatment. Simulated patients in OSCE stations will also assess each student on empathic engagement using the 5-item JSPPPE (Jefferson Scale of Patient Perception of Physician Empathy) [37]. Results of this assessment will be provided to the students at the end of the second training session in the form of individual oral feedback, as students in the pilot teaching sites indicated OSCE practice and individual feedback was valuable in improving their own skills and exam performance. It will also be made clear to the students that the OSCE in READ is for training, and not a formal examination of their abilities, to reduce any potential anxiety the students may associate with OSCEs in general. To increase reliability and comparability of the results the sites have been provided with the OSCE scenario and a standardised marking scheme describing the objectives and assessment process. In addition, where possible, examiners will not know whether participants are in the intervention or the control group. Although the OSCE may be perceived as formal, the structured and uniform nature of this type of assessment provides an objective and standardised instrument to measure change in skills, a key new area that this study seeks to evaluate.

### Empathy

The Medical Student version of the Jefferson Scale for Empathy (JSE-S) is a 20 item scale with well-established psychometric properties [38, 39] and is available in multiple languages. Each item has a 7 point Likert scale and the total score ranges 7–140.

### Intergroup anxiety

We will employ Stephan and Stephan’s 12 item intergroup anxiety measure modified for medical students [40]. The scale asks respondents to rate how much they experienced a range of feelings (anxious, apprehensive, comfortable, secure, worried, calm, confident, awkward, tense, carefree, nervous, and at ease) from 0 = not at all to 4 = extremely. A score is created by reverse scoring the positive feelings and averaging all the items.

### Sample size

Using the MAKS, the overall change in score at the first follow up point in a previous study [14] using a medical student sample was 1.91. The standard deviation for the MAKS varies among samples but is typically below 3. Using a standard deviation of 2.89 from one sample gives an effect size of 0.66, and including a dropout rate of 10%, a power level of 90% is achieved with a sample size of 448 in each group. This sample size will be achieved if each of the 25 sites recruits on average 36 students, with group sizes depending on local teaching arrangements. We are anticipating that some sites will recruit larger samples and others smaller, according to the available resources and size of the medical schools.

### Recruitment

Eligible medical students will be those currently undertaking their rotational training in Psychiatry, which takes place in different years of training in different countries. An Information Sheet to eligible medical students will be distributed at least 24 h before the first session via email or by hand at a previous teaching session, and again at the start of the first session; written informed consent will then be sought at the start of the first session. One to one consent will be ensured through the availability of multiple members of the research team who will answer individual students’ queries. Non-participating students will not be able to attend the training, as their receipt of the training without providing data may be perceived as unfair by participating students. It will be made clear to students prior to and at the start of the session that non-participation will not affect students’ future performance on their medical course in any way.

### Procedure for participants and data collection

**Fig. 1:**
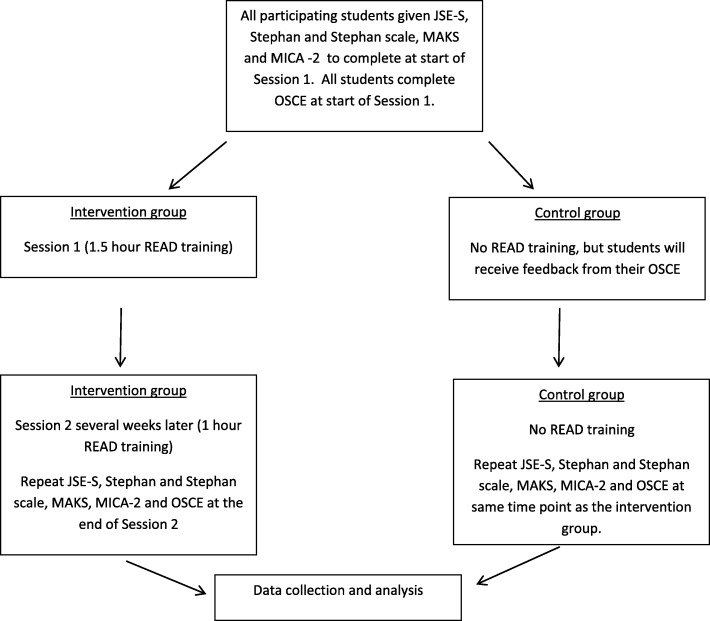
Flow diagram to illustrate the process for study participation

Once written informed consent has been received, participating students in the intervention group will be given the questionnaire based measures to complete, and will then undertake the OSCE at the start of Session 1. Data collection using the questionnaire based measures and the OSCE will be repeated at the end of Session 2. Students in the control groups will be given the same measures and will carry out the OSCE during the same time frame as the intervention group. Instead of receiving the intervention they will be able to discuss the OSCE feedback with the role player, after the second attempt (Fig. 1).

### Data management

Data will be entered locally using a database provided by the research group at the lead site at King’s College London. Pre and post data will be linked using a study ID number and no identifying information will be included. The cleaned, final dataset at each site will be returned to the lead at the King’s College London site for analysis of the whole sample (both intervention and controlled arm). Data collection will be completed by the end of 2018.

All data collection and data entry will be performed by trained research staff. If errors are found in the database, inconsistencies will be clarified by referring to the relevant hard copy. The quality of data collection will be supervised by senior researchers in the site, with overall supervision by the principal investigator. A Microsoft Excel database has been developed to include data type checks and field guidance. Supervisors will monitor data quality, periodically carry out a set of pre-defined quality checks on the data, raise any queries with the relevant staff and identify missing data in the field. Where data are found to be missing, researchers will endeavour to revisit the participant to obtain the missing data. All storage of data will be in accordance with King’s College London’s data protection policy (http://www.kcl.ac.uk/college/policyzone/assets/files/governance_and_legal/DataProtectionPolicy_updated_Oct2011.pdf). All data will be anonymised for analysis and will be accessible to the project investigators, the project co-ordinator and the data entry clerks within the INDIGO Partnership. Identifying data will be kept as a separate list with identifying codes, for the research records. The document linking the project identification number with personal data will only be accessible to the study research team in the country sites, restricted to the project co-ordinator and data entry clerks, and all the anonymised data will be used for analyses.

### Statistical analysis

Total scores for MAKS, MICA2 and the OSCE will be calculated so that a higher total score represents a more favourable outcome. Descriptive summaries of score changes will be calculated between the baseline and follow up time points for each group. Parametric and non-parametric tests will be employed to detect statistically significant differences in means on individual-level data before and after implementation of the intervention compared to the controls. Statistical analyses of individual-level data will be conducted using a random effects model accounting for clustering within sites and countries, to take into account the between country and culture variation in the attitudes towards mental illness. Changes in each outcome will be investigated at baseline and at the follow up time point after the intervention. All models will be adjusted for gender; age (as a continuous variable); country income category (high, middle or low) and familiarity with people with mental health problems (self or other i.e. family member, friend). In order to appropriately quantify the direct and indirect effects of empathy and anxiety on the three outcomes (MAKS, MICA2 and the OSCE), we will conduct a mediation analysis under the causal framework with the use of the g-computation formula, which has been shown to produce reliable estimates in such scenarios (http://aje.oxfordjournals.org/content/early/2014/12/10/aje.kwu239.full.pdf).

### Process evaluation

This study will follow guidance from the UK Medical Research Council on assessing the context, implementation and mechanisms of action. Site leads will be asked to provide relevant background information which may affect the implementation of the intervention, including: length of the Psychiatry rotation; year of degree course of in which the Psychiatry rotation takes place; whether OSCEs are routinely used in medical education and the psychiatry teaching curriculum.

Students’ attendance will be noted using sign in and sign out sheets for each session, asking for the time of the signature. The total number of students at each site and the proportion lost to follow up will also be recorded in the database of results.

As part of fidelity assessment delivery of each component of the intervention will be monitored by a member of the research team at each site using a checklist. The checklist will also include prompts to describe barriers to delivering any components. Remote supervision and support for site leads will be provided on request by the KCL research team as needed.

## Discussion

This study applies components found to be effective from medical education research in an anti- stigma training intervention for medical students. It incorporates reflection and self reflection, which has been effective in consolidating learning and improving attitudes in medical education studies [15]. This is particularly relevant as the intervention aims to change attitudes in medical students towards patients with mental illness. The OSCE involves communication and interviewing skills training, and previous work in medical education demonstrated good communication skills lead to greater satisfaction with care in patients [41] In addition studies have shown a decrease in patients’ distress and potential susceptibility to symptoms of depression or anxiety, in response to good interviewing skills [42]. Therefore training students in interviewing skills should lead to patients feeling more able to express concerns regarding anticipated and experienced discrimination in clinical consultations.

This is also the first international study across high, middle and low-income countries, which will evaluate the effectiveness of training for medical students to respond to experienced and anticipated discrimination. The range of sites will provide a valuable contribution to test the feasibility and effect of the training in multiple settings, therefore increasing the validity of any relevant conclusions drawn from the results. In addition this study measures change in skills at addressing discrimination using an OSCE, which is a new contribution to the field of stigma research. Previous studies have mostly focused on measuring change in knowledge and attitudes [11, 12].

As the study is unfunded there is a risk some sites may drop out. In this event we will ask the remaining sites to increase their sample numbers if possible, to achieve the target sample size. Several sites (including the UK) have resources available to do so.

Another possible limitation of the study is self-selection bias – i.e. students who already have less stigmatising attitudes and behaviour may be more likely to participate in the study, and may wish to be in the intervention group. Sites will therefore assign medical students using existing teaching groups to either the intervention or control group. To encourage control students to still attend and to minimise drop out rates, the OSCE examiner will provide oral feedback at the end of the second OSCE, which students may find useful for their general medical training and their future examinations [29].

The results of this study will help medical schools in high, middle and low income countries to implement manualised training to enable future doctors to reduce the effects of mental illness related discrimination on their patients.

## Abbreviations

JSE-S: Jefferson Scale for Empathy, medical student version
JSPPPE: Jefferson Scale of Patient Perception of Physician Empathy
MAKS: Mental Health Knowledge Schedule
MICA-2: Mental Illness Clinicians’ Attitude Scale, medical student version
READ: Responding to Experienced and Anticipated Discrimination

## References

[1] Clement S, Schauman O, Graham T, et al. What is the impact of mental health-related stigma on help-seeking? A systematic review of quantitative and qualitative studies. Psychol med 45:11–27. Doi. 2015. 10.1017/S0033291714000129. 10.1017/S0033291714000129 24569086

[2] Corrigan PW, Druss BG, Perlick DA (2014). The impact of mental illness stigma on seeking and participating in mental health care. Psychol Sci Public Interes.

[3] Suhrcke M, de Paz Nieves C (2011) The impact of health and health behaviors on educational outcomes in high-income countries: a review of the evidence. WHO regional Office for Europe, Copenhagen, DK.

[4] Lee S, Tsang A, Breslau J (2009). Mental disorders and termination of education in high-income and low and middle-income countries: epidemiological study. Br J Psychiatry.

[5] Mai Q, Holman CDJ, Sanfilippo FM, et al. Mental illness related disparities in diabetes prevalence, quality of care and outcomes: a population-based longitudinal study. BMC Med. 2011. 10.1186/1741-7015-9-118. 10.1186/1741-7015-9-118 PMC3215928 22044777

[6] Laursen TM, Munk-Olsen T, Nordentoft M, Mortensen PB (2007). Increased mortality among patients admitted with major psychiatric disorders: a register-based study comparing mortality in unipolar depressive disorder, bipolar affective disorder, schizoaffective disorder, and schizophrenia. J Clin Psychiatry.

[7] Gissler M, Laursen TM, Oesby U, et al. Patterns in mortality among people with severe mental disorders across birth cohorts: a register-based study of Denmark and Finland in 1982-2006. BMC Public Health. 2013. 10.1186/1471-2458-13-834. 10.1186/1471-2458-13-834 PMC3850635 24025120

[8] Department of Health (2011) No health without mental health. HM Government, London, UK.

[9] World Health Organisation (2013) Mental Health Action Plan 2013–2020. doi: 10.1017/CBO9781107415324.004

[10] Henderson C, Noblett J, Parke H, Clement S, Caffrey A, Gale-Grant O (2014). Mental health-related stigma in health care and mental health-care settings. Lancet Psychiatry.

[11] Knaak S, Modgill G, Patten SB (2014). Key ingredients of anti-stigma programs for health care providers: a data synthesis of evaluative studies. Can J Psychiatry.

[12] Sartorius N (1998). Stigma: what can psychiatrists do about it?. Lancet.

[13] Kassam A, Glozier N, Leese M, Loughran J, Thornicroft G (2011). A controlled trial of mental illness related stigma training for medical students. BMC medical education.

[14] Friedrich B, Evans-Lacko S, London J, Rhydderch D, Henderson C, Thornicroft G (2013). Anti-stigma training for medical students - the education not discrimination project. Br J Psychiatry.

[15] Fragkos KC (2016). Reflective practice in healthcare education: an umbrella review. Educ Sci.

[16] Mezirow J (1981). How Critical Reflection triggers Transformative Learning.

[17] Bekas S (2013). Critical reflection: a sound foundation for learning and practice in psychiatry. Adv Psychiatr Treat.

[18] Gronholm PC, Henderson C, Deb T, Thornicroft G. Interventions to reduce discrimination and stigma: the state of the art. Social Psychiatry and Psychiatric Epidemiology 2.1.2017. DOI 10.1007/s00127-017-1341-9. 10.1007/s00127-017-1341-9 PMC5344948 28144713

[19] Thornicroft G, Mehta N, Clement S, Evans-Lacko S, Doherty M, Rose D, Koschorke M, Shidhaye R, O’Reilly C and Henderson C. Evidence for effective interventions to reduce mental health related stigma and discrimination: narrative review. The Lancet, published online September 23, 2015 10.1016/S0140-6736(15)00298-6. 10.1016/S0140-6736(15)00298-6 26410341

[20] Mehta N, Clement S, Marcus E, Stona A-C, Bezborodovs N, Evans-Lacko S, Palacios J, Doherty M, Barley E, Rose D, Koschorke M, Shidhaye R, Henderson C, Graham TG (2015). Systematic review of evidence for effective interventions to reduce mental health related stigma and discrimination: medium and long-term effectiveness. Br J Psychiatry.

[21] Al Ramiah A, Hewstone M (2013). Intergroup contact as a tool for reducing, resolving, and preventing intergroup conflict: evidence, limitations, and potential. Am Psychol.

[22] Yamaguchi S, Mino Y, Uddin S (2011). Strategies and future attempts to reduce stigmatization and increase awareness of mental health problems among young people: a narrative review of educational interventions. Psychiatry ClinNeurosci.

[23] Stuart H (2014). Opening minds in Canada: background and rationale. Can J Psychiatr.

[24] Stuart H (2014). Opening minds in Canada: targeting change. Can J Psychiatr.

[25] Corker E, Hamilton S, Robinson E, Cotney J, Pinfold V, Rose D, Thornicroft G, Henderson C (2016). Viewpoint survey of mental health service users’ experiences of discrimination in England 2008–2014. Acta Psychiatr Scand.

[26] Thornicroft G, Brohan E, Rose D, Sartorius N, Leese M, INDIGO Study Group (2009). Global pattern of experienced and anticipated discrimination against people with schizophrenia: a cross-sectional survey. Lancet.

[27] Lasalvia A, Zoppei S, Bortel TV, Bonetto C, Cristofalo D, Wahlbeck K, Bacle SV, Audenhove CV, van Weeghel J, Reneses B, Germanavicius A, Economou M, Lanfredi M, Ando S, Sartorius N, Lopez-Ibor J, Thornicroft G, the ASPEN/INDIGO study group (2013). Global pattern of experienced and anticipated discrimination reported by people with major depressive disorder: a cross-sectional survey. Lancet.

[28] Schulze B, Angermeyer MC (2003). Subjective experiences of stigma. A focus group study of schizophrenic patients, their relatives and mental health professionals. Soc Sci Med.

[29] Hamilton S, Pinfold V, Cotney J, Couperthwaite L, Matthews J, Barret K, Warren S, Corker E, Rose D, Thornicroft G, Henderson C (2016). Qualitative analysis of mental health service users’ reported experiences of discrimination. Acta Psychiatr Scand.

[30] J Noblett, A Caffrey, T Deb, A Khan, E Lagunes-Cordoba, O Gale-Grant, C Henderson liaison psychiatry professionals’ views of general hospital care for patients with mental illness: the care of patients with mental illness in the general hospital setting. J Psychosom Res 95 (2017) 26–32. 10.1016/j.jpsychores.2017.02.004 28314546

[31] Shefer G, Henderson C, Howard LM, Murray J, Thornicroft G. Diagnostic overshadowing and other challenges involved in the diagnostic process of patients with mental illness who present in emergency departments with physical symptoms – a qualitative study. PLoS One. 2014;9(11):e111682. 10.1371/journal.pone.0111682. 10.1371/journal.pone.0111682 PMC4219761 25369130

[32] West K, Holmes E, Hewstone M (2011). Enhancing imagined contact to reduce prejudice against people with schizophrenia. Gr Process Intergr Relations.

[33] Pettigrew TF, Tropp LR (2008). How does intergroup contact reduce prejudice? Meta-analytic tests of three mediators. Eur J Soc Psychol.

[34] Evans-Lacko S, Little K, Meltzer H, Rose D, Rhydderch D, Henderson C (2010). Development and psychometric properties of the mental health knowledge schedule. Can J Psychiatry.

[35] Kassam A, Glozier N, Leese M, Henderson C, Thornicroft G (2010). Development and responsiveness of a scale to measure clinicians' attitudes to people with mental illness (medical student version). Acta Psychiatr Scand.

[36] Khan KZ, Ramachandran S, Gaunt K, Pushkar P (2013). The objective structured clinical examination (OSCE): AMEE guide no. 81. Part I: an historical and theoretical perspective. Med Teach.

[37] Hojat M, Louis DZ, Maxwell K, Markham F, Wender R, Gonnella JS (2010). Patient perceptions of physician empathy, satisfaction with physician, interpersonal trust, and compliance. Int J Med Educ.

[38] Hojat M, Gonnella JS (2015). Eleven years of data on the Jefferson scale of empathy-medical student version (JSE-S): proxy norm data and tentative cutoff scores. Med Princ Pract.

[39] Hojat M, LaNoue M (2014). Exploration and confirmation of the latent variable structure of the Jefferson scale of empathy. Int J Med Educ.

[40] Stephan WG, Stephan CW (1985). Intergroup anxiety. J Soc Issues.

[41] Clever SL, Jin L, Levinson W, Meltzer DO (2008). Does doctor-patient communication affect patient satisfaction with hospital care? Results of an analysis with a novel instrumental variable. Health Serv Res.

[42] Roter DL, Hall JA, Kern DE, Barker LR, Cole KA, Roca RP (1995). Improving physicians’ interviewing skills and reducing patients' emotional distress. Arch Intern Med.

